# Extruded Preparations with Sour Cherry Pomace Influence Quality and Increase the Level of Bioactive Components in Gluten-Free Breads

**DOI:** 10.1155/2020/8024398

**Published:** 2020-07-01

**Authors:** Dorota Gumul, Anna Korus, Rafał Ziobro

**Affiliations:** ^1^Department of Carbohydrates Technology, Faculty of Food Technology, University of Agriculture in Krakow, Balicka 122 Street, 30-149 Krakow, Poland; ^2^Department of Plant Product Technology and Nutrition Hygiene, University of Agriculture in Krakow, Balicka 122 Street, 30-149 Krakow, Poland

## Abstract

Gluten-free bread (GFB) usually has a lower nutritional value than its traditional counterparts and is deficient in health-promoting substances. Therefore, GFB is often enriched in gluten-free components containing high levels of bioactive substances. In this work, an attempt has been made to enrich GFB with rice flour-based extruded preparations produced at 80 and 120°C with a share of 10 and 20% sour cherry pomace. The study material consisted of the abovementioned preparations together with breads produced with their 10% share. In order to prove that the extruded preparations could be the source of phenolic compounds, their level was determined. The influence of the applied additions was assessed taking into account nutritional composition (protein, fat, ash, and carbohydrates), level of the phenolic compounds (total phenolic content, flavonoids, anthocyanins, and phenolic acids), antioxidant potential, and physical properties of the breads (texture volume, color). It was shown that the extrudates with a share of fruit pomace cause an enrichment of gluten-free breads in bioactive compounds. The gluten-free breads enriched in extrudates with sour cherry pomace obtained at 120°C contained even 6 times more polyphenols than breads with extrudates obtained at 80°C. At the same time, these breads contained the highest levels of flavonoids and phenolic acids among all the analyzed samples. Bread with the addition of the extrudate produced with 20% fruit pomace at 120°C was the most favorable in terms of bioactive compounds (total phenolic content, flavonoids, anthocyanins, and phenolic acids) and antioxidative activity. The abovementioned bread showed the highest amount of total, soluble and insoluble fiber, and a significant amount of ash and sugars and revealed the lowest hardness during 3 days of storage, in comparison with the other samples.

## 1. Introduction

Strict elimination of cereals and grain products containing gluten from the diet seems to be the only efficient treatment of celiac disease. Although the prolonged use of such diet by persons without celiac disease is doubtful [1], the number of people choosing it without any medical background is growing [2]. Considering the fact that gluten-free products are usually poor in nutritional value and health-promoting substances, their unbalanced use may induce other disorders, i.e., osteoporosis, rickets, anemia, and slowdown of mental and physical development [2]. Therefore, special attention should be paid to enrichment of such products in raw materials which are a source of prohealth components. In this context, new recipes should be developed which would broaden the portfolio of available gluten-free products and minimize the risk of nutritional deficiencies for celiacs and other people adhering to gluten-free diet.

There is a number of possibilities for enriching gluten-free bread, ranging from addition of pseudocereals, oilseeds, vegetables, and fruits to the use of by-products manufactured by food processing industry [3–12]. One of the additives with a high potential for providing a broad range of prohealth substances is fruit pomace. Sour cherry (*Prunus cerasus* L.) is an important type of fruit yielding large quantities of pomace. Annual yield of sour cherry in Poland is high, and the fruit is processed into juices, nectars, soft and alcoholic drinks and jams, which is accompanied by the formation of this by-product. Sour cherry pomace is a rich source of anthocyanins, hydroxycinnamic acids (neochlorogenic, chlorogenic, dicaffeoylquinic acids, and p-coumaroylquinic acids) dimer, trimer, tetramer procyanidin, quercetin, kaempferol, and also vitamins, mineral compounds, and dietary fiber (cellulose, hemicellulose, and pectin) [13–22]. Therefore, sour cherry pomace containing large quantities of bioactive and nutritive compounds seems to be a valuable and very cheap component in a production of gluten-free bread. It would allow to reduce the quantity of stored by-products. It should be remembered that the abovementioned bioactive compounds act chemopreventively on human health. Antiallergic, anticancer, antiviral, and antibacterial activity of these compounds will largely depend on their bioavailability [13, 16]. Bioavailability of these constituents can additionally be altered by appropriate modifications, *e.g.*, by physical means. Extrusion seems to be especially suitable as a method, because it allows an immediate conversion of microbiologically unstable raw material into a sterile, dry component, which could be easily stored, ground, and added into various food matrices. Additionally, such processing is known to result in changes of dietary fiber into more soluble forms, highly beneficial for human health. In the case of bioactive polyphenols, extrusion could cause their decrease due to decomposition, or conversely, their increase by the release from fiber, making them more bioavailable, depending on the parameters of extrusion [16].

The use of fruit pomace in the form of extrudates for the production of gluten-free bread is innovative. It should be kept in mind that bioactive compounds present in sour cherry pomace are thermally labile during processing. The application of extrusion of fruit pomace with rice flour results in encapsulation of these compounds in starch matrix which results in their protection against thermal destruction during baking and promotes bioavailability during digestion.

The aim of the study was to analyze the influence of extruded sour cherry pomace–rice flour preparations (ECPRF) on the nutritional, prohealth components of gluten-free bread. The product was also checked in terms of physical properties, because they are especially important for potential consumers.

## 2. Materials and Methods

### 2.1. Materials

In the initial stage of the experiment, the extrudates were obtained in a single screw laboratory extruder Brabender 20 DN (Duisburg, Germany) at 80 and 120° from rice flour with no addition, as well as 10 and 20% (*w*/*w*) share of sour cherry pomace. The extrusion conditions were as follows: screw speed 190 r.p.m., die diameter 4 mm, compression ratio 1 : 3; moisture level of all extruded premixes was equilibrated to 14%. Rice flour used for extrusion contained 5.37% protein, 2.68% fat, 0.68% ash, and 91.5% starch, while sour cherry pomace 13.57% protein, 3.02% fat, 10.97% carbohydrates, and 48.7% dietary fiber (4.5% soluble, 44.2% insoluble). Extrusion temperatures were set so as to differentiate the levels of bioactive compounds in the extrudates. The level of sour cherry pomace was limited in order to provide the acceptable color of the bread. Sour cherry pomace (*Prunus cerasus L*.) was obtained from ZPOW Hortino sp. z o.o. (Leżajsk, Poland), while rice flour (Look Food company) was purchased from a local store.

The preparations were identified as 10/80, 20/80, 10/120, 20/120 (i.e., extruded preparation based on rice flour with 10% fruit pomace obtained at extrusion temperature 80°C, and similarly other labels), and 0/80 and 0/120 (i.e., extruded preparation based on rice flour obtained at extrusion temperature 80°C without fruit pomace and similarly the other label).

In the second stage of the experiment, the research material consisted of gluten-free breads with 10% share of the extrudates produced with or without sour cherry pomace. Materials for baking gluten-free breads included also corn starch (Bezgluten, Poland), potato starch (PEPEES S.A., Poland), guar gum (Lotus Gums & Chemicals, India), pectin (Pektowin, Poland), freeze-dried yeast (S.I. Lesaffre, France), sucrose, salt, and canola oil (the latter three ingredients were bought in local stores).

Breads were identified in the text, tables, and figures as K control bread, SBK 0/80, and SBK 0/120, starch bread with 10% rice extrudate processed at 80 and 120°C without fruit pomace, respectively; SB10/80, SB10/120, starch bread with 10% rice extrudate containing 10% fruit pomace, processed at 80 and 120°C, respectively; and similarly other labels.

### 2.2. Bread Preparation

The basic recipe for control bread based on Witczak et al. [23] used the following amounts of ingredients: a mixture of maize starch and potato starch in ratio 4 : 1 600 g, guar gum 10 g, pectin 10 g, freeze-dried yeast 30 g, sucrose 12 g, salt 11 g, rapeseed oil 18 g, water 570 g. Part of the starch mixture (10%, i.e., 50 g) was replaced by the tested extrudates. All components were mixed for 5 min (Laboratory Spiral Mixer SP 12, Diosna, Germany). The dough was fermented for 15 min (35°C, 80% moisture), remixed for 1 min, and 200 g dough pieces were put into the greased molds. The final fermentation, under the conditions described above, lasted 20 min. The breads were baked in a MIWE Condo oven type CO-2-0608 (MIWE GmbH, Germany) for 30 min at 240°C upper heater and 210°C lower heater. Two dough samples were made according to each recipe (two independent repetitions); then, six breads were baked, separately from each dough. The loaves for physical evaluation during the following days were packed in polyethylene bags and stored in a chamber at 20 ± 2°C, relative humidity 64%. Bread crumbs for chemical and nutritional evaluation were dried at room temperature, ground, sifted through a 1 mm^2^ mesh screen and stored in glass jars.

### 2.3. Nutritional Evaluation of Starch Breads with a Share of ECPRF

Content of basic nutritional components (protein, fat, total carbohydrates, ash, and moisture) was performed by the methods of AOAC [24]. The content of protein was determined by the Kjeldahl method AOAC No. 950.36 (using the extraction system Büchi B324, Nx5.7; Büchi Labortechnik, Flawil, Switzerland), fat by the Soxhlet method AOAC No. 935.38 (using Büchi B811; Büchi Labortechnik), total carbohydrates by AOAC No. 974.06, ash by AOAC No. 923.03, and moisture by AOAC No. 926.05. The content of dietary fiber was determined by the method 32-07 of AACC [25]. All the measurements were done at least in duplicate.

### 2.4. Chemical Evaluation of Preparation ECPRF and Starch Breads with a Share of ECPRF

Antioxidant constituents and antiradical activity were determined in the ethanol extracts. Determination of total polyphenols content (TPC) was done by spectrophotometric methods using Folin-Ciocalteu reagent, according to Singleton et al. [26]. The content of phenolic acids and anthocyanins was measured using a spectrophotometrical method, according to Mazza et al. [27], with the modification of Oomah et al. [28]. The content of flavonoids was evaluated using a spectrophotometrical method, according to El Hariri et al. [29]. Additionally antiradical activity was assessed using analytical methods with ABTS (2,2′-azino-bis(3-ethylobenzothiazoline-6-sulphonic acid)-diamonium salt) [30]. All the measurements were done at least in duplicate.

### 2.5. Physical Evaluation of Starch Breads with a Share of ECPRF

The volume of the loaves was measured using a Volscan profiler 600 laser meter (Stable Micro Systems, England).

Color parameters of the crumb of analyzed breads were determined using 1 cm thick slices cut from the analyzed loaves using an instrumental method in a system CIE (*L*∗, *a*∗, and *b*∗). Color components were acquired using the reflection method by Konica MINOLTA CM-3500d. The angle of measurement was 10°. A 30 mm diaphragm and a 55 mm diameter Petri dish were used for the measurement.

The texture profile analysis (TPA) measurements of the tested loaves were made during 3 days of storage using a TA-XT-plus texture analyzer (Stable Micro Systems, England) [31]. The test sample was taken from a 2 cm thick slice obtained from the center of the loaf and compressed by P/20 aluminum cylinder probe. The standard program was applied, using a compression rate 5 mm s^−1^, deformation rate 50%, and a delay between two cycles 5 s. The results were calculated with the Texture Exponent program (Stable Micro Systems, England).

Image analysis is as follows: bread slice of the thickness 1 cm, cut from the center of the loaf, was scanned using the Plustek S-12 desktop scanner. The images were analyzed using ImageJ software v. 1.44c [32]. While performing physical evaluation, three loaves from each batch (6 replications) were analyzed, except for TPA, in which case two loaves from each batch were taken on each day (4 replications).

### 2.6. Statistical Analysis

The obtained data were analyzed by one-factor or two-factor (texture analysis: first factor, formulation; second factor, time) analysis of variance, and the least significant difference (LSD) at significance level 0.05 was calculated with Statistica 10.0 (StatSoft Inc., USA). The Pearson correlation coefficients between selected parameters were also calculated.

## 3. Results and Discussion

### 3.1. Characteristics of Extruded Sour Cherry Pomace-Rice Flour Preparations (ECPRF)

Extruded preparations based on rice flour with fruit pomace (10 and 20%) processed at 80 and 120°C are new functional ingredients for gluten-free bread enrichment. The extrusion used in the production of these preparations may cause encapsulation of bioactive compounds contained in food matrix, in this case sour cherry pomace. The research conducted so far was focused only on the encapsulation of individual bioactive substances, natural, or synthetic ones (*e.g.*, gallic acid, catechin, caffein, aminoacids, and isoflavons) [33, 34], rarely using food matrices.

Encapsulation of functional compounds in starch material by the extrusion process is gaining increasing interest due to not only low production costs and environmentally friendly technology [35, 36] but also the possibility of improving bioavailability of the bioactive component during human digestion [33, 37]. It can be said that the process of encapsulation will be the added value of the analysed preparation, which will serve to enrich the products (*e.g.*, gluten-free breads) with bioactive prohealth compounds, while ensuring their high bioavailability.

Total polyphenol content (TPC) in 10/80, 20/80, 10/120, and 20/120 preparations was 21.2, 48.5, 59.95, and 86.3 mg catechin/100 g d.m., respectively. It was found that the elevation of extrusion temperature resulted in an increase in the content of polyphenols. The amount of flavonoids in 10/80, 20/80, 10/120, and 20/120 preparations was 3.9, 5.64, 7.28, and 19.05 mg rutin per 100 g d.m, respectively. The amount of flavonoids increased 2 to 4 times in the preparations along with the rising extrusion temperature. This trend is most probably due to the fact that elevated temperature increases pressure and shear forces in extruder's barrel which leads to liberation of phenolic compounds, including phenolic acids, from complexes formed with dietary fiber forming cell walls. Therefore, the amount of phenolic compounds, including phenolic acids, is higher and their bioavailability is improved [13–22]. In the study of Bisharat et al. [13] concerning extrusion of maize flour with broccoli, it was observed that the increase in extrusion temperature positively influenced phenolic compounds. According to Zhang et al. [14], extrusion of rye at 120°C caused higher loss of polyphenols that the process performed at 180°C. The authors also explain this tendency by the release of polyphenols from fiber complexes, changing the conjugated or bound form to free form, which increases their extractability, especially from the material obtained at higher extrusion temperatures. Leyva-Corral et al. [15] proved that higher extrusion temperature could lead to the preservation of bioactive compounds or even cause their increase, which is confirmed by the results presented here. The amount of anthocyanins in 10/80, 20/80, 10/120, and 20/120 preparations was 1.97, 2.57, 4.7, and 5.3 mg cyanidin-3-glucoside per 100 g d.m., respectively. Phenolic acids were only present in preparations obtained at 120°C, and their level equaled 1.6 and 2.6 mg ferulic acid per 100 g d.m. when of 10 and 20% of cherry pomace were applied, respectively. The correlation coefficient between TPC and content of flavonoids, anthocyanins, and phenolic acids equaled 0.900, 0.922, and 0.903, respectively.

In the case of extrudates produced without sour cherry pomace (0/80, 0/120) no bioactive compounds were detected, because their presence was possible only in the fruit component. The ECPRF (with 10 and 20% share of sour cherry pomace) produced at extrusion temperatures 80 and 120°C contain a significant amount of bioactive compounds and may constitute new functional components for gluten-free bread enrichment, not used in the production of gluten-free bread earlier.

### 3.2. Chemical Composition of Bread Samples

Visible increase in reducing sugars could be observed due to partial replacement of starch in basic recipe with ECPRF (Table 1). Due to a low initial level of these compounds (0.1%), the magnitude of this increase was quite significant (6-7-fold). The appearance of reducing sugars is due to the introduction of starch-based extrudates, which contain significant amounts of oligosaccharides, produced by starch decomposition during extrusion. The application of ECPRF had a marginal influence on basic constituents, such as protein and fat. Some increase could be observed in mineral content, accompanied by a similar decrease in starch content (Table 1). The increase in ash was observed in gluten-free bread with ECPRF preparations because sour cherry pomace is a good source of minerals. The use of rice flour extrudates without sour cherry pomace for gluten-free bread preparation did not result in changes in ash content (SBK 0/80, SBK 0/120). The decrease in starch in bread was significant in which sour cherry pomace extrudates were used, but not in those with pure rice flour extrudates (except SBK 0/80). This reflects the decrease in total starch content caused by its partial replacement with fruit component. Small but statistically significant decrease of starch content in SBK 0/120 bread as compared to control may be caused by partial complexing of this component with proteins and fat present in rice flour, especially at a higher extrusion temperature [38].

More obvious trends could be seen for fractions of dietary fiber (Table 2). While basic formulation (control bread) resulted in bread containing low amounts of its soluble fraction, each of the applied rice extrudates with or without sour cherry pomace caused an increase in its level. The increase in the soluble fiber fraction in breads with the extrudates ranged between 24 and 36% in relation to control. The opposite trend could be seen for insoluble fraction. Its level was reduced after the addition of extrudates produced without the share of the pomace, most probably due to the replacement of native, granular starch with gelatinized starch (present in rice flour extrudates), which was less likely to form insoluble complexes. These changes were reversed when fruit component was included in the extrudates, and the final level of insoluble dietary fiber in bread with the addition of extrudates with 20% pomace was comparable or even higher to control. Both these effects were responsible for the final value of total dietary fiber (TDF) which was the highest in the samples containing extrudates produced with 20% pomace, especially those processed at higher extrusion temperature (Table 2). In the case of the SB20/80 sample, the increase in TDF was 7.5%, while for SB20/120, it reached 15% in comparison to control. According to Tsatsaragkou et al. [20], 15% addition of carob germ to a rice-based gluten-free formulation caused an increase in TDF by 6.1% and protein by 8.4% in comparison to control. In the study of Korus et al. [12] gluten-free bread enriched with defatted strawberry and blackcurrant seeds contained 33 to 120% more protein in comparison to control. In the case of soluble, insoluble, and total dietary fiber, their increase in gluten-free bread caused by the application of defatted strawberry and blackcurrant seeds was 3-, 2-, and 2,5-fold in comparison to control, respectively. O'Shea et al. [19] observed doubling the content of fiber in gluten-free bread after the addition of 5.5% orange pomace.

### 3.3. Bioactive Compounds of Bread Samples

Table 3 presents total polyphenol content (TPC) in starch-based breads with the addition of ECPRF. It was found that the starch bread which was used as the control did not contain the abovementioned compounds. At the same time, it was shown that extruded preparations with or without added fruit pomace caused the appearance of polyphenols in gluten-free bread, the exception in this respect was extruded rice flour obtained at a temperature of 120°C. It should be underlined that the presence of TPC in bread SBK0/80 is due to the formation of Maillard reaction products, which could react with Folin-Ciocalteu's reagent used to evaluate the polyphenols in the applied method. According to Shahidi and Naczk [39] as well as Gallardo et al. [40]. Folin-Ciocalteau's reagent interacts with other components, e.g., Maillard reaction products, overstating the result. The observed, elevated level of TPC in the abovementioned sample is therefore apparent as the real polyphenols are introduced by the addition of extrudates with a share of sour cherry pomace. Of all, analyzed loaves with the addition of extruded preparations bread with the participation of rice extrudates with a 20% share of fruit pomace obtained at extrusion temperature of 120°C were characterized by the greatest content of polyphenols, and one with the rice flour extrudate, obtained at 80°C by their smallest level. The highest TPC in SB20/120 (30.87 mg catechin/100 g d.m.) is caused by the largest content of these compounds in extruded preparation 20/120 (86.27 mg catechin/100 g d.m.).

It was clearly visible that gluten-free breads baked with ECPRF obtained at 120°C contained even six times more polyphenols than those obtained with the addition of ECPRF extruded at 80°C. This trend could be explained by the fact that extrudates with a share of sour cherry pomace which were obtained at 120°C display larger values of TPC, because as it was earlier observed, more phenolic compounds are freed at high extrusion temperatures. As a consequence, the application of these extrudates for gluten-free bread formulations results in higher polyphenol content in comparison to bread with extrudates obtained at 80°C. In the studies conducted by Kruczek et al. [41] on gluten-free breads with the addition of dried apple pomace, plant material caused an increase in polyphenol content compared to the control. Also in the studies of Constantini et al. [5], focused on wheat and gluten-free bread with chia seeds, a 30% increase in polyphenol content was observed after the addition of the seeds to bread formulation. Also Gumul et al. [8] observed a two- to fivefold increase in total polyphenols in gluten-free breads with the addition of dried red potatoes in relation to the control. Korus et al. [12] analyzing the quality and content of polyphenols and antioxidant activity in gluten-free breads with the participation of defatted blackcurrant and strawberry seeds noticed an increase in the content of these ingredients ranging from 92% to 130% compared to the control.

Basing on the results shown in Table 4, it was found that breads with extruded rice flour as well as those containing preparations with 10% and 20% share of fruit pomace processed at 80°C did not show the presence of phenolic acids. Their presence was only recorded in breads which were supplemented with ECPRF obtained at 120°C. This proves that phenolic acids are present only in the extrudates containing sour cherry pomace obtained at the highest applied extrusion temperature. Considering the content of flavonoids, the highest level of these compounds was found in starch-based bread with the addition of rice extrudate containing 20% share of fruit pomace obtained at 120°C, and the smallest in starch bread with 10% addition of rice extrudate containing 10% share of fruit pomace obtained at 80°C. At the same time, it was noticed that ECPRF obtained at a higher temperature (120°C) provided several times higher content of flavonoids in gluten-free breads than those processed at a lower temperature (80°C). The presence of anthocyanins in control and gluten-free breads baked with the addition of rice flour extruded at 80°C and 120°C was not detected. Anthocyanins appeared in breads, which were baked with the participation of ECPRF, because it introduced large amounts of these compounds into gluten-free dough. The content of anthocyanins in breads with the participation of fruit pomace processed at 80°C was smaller (on average by 12%) than in breads with the addition of preparations extruded at 120°C. The largest amount of anthocyanins was found in bread with the addition of an extrudate obtained at 120°C with a 20% share of fruit pomace (Table 4). Such a high level of anthocyanins in bread was caused by their presence in ECPRF 20/120 (5.3 mg cyanidin-3-glucoside/100 g d.m.).

The content of bioactive compounds in starch based bread is a favorable feature resulting from the introduction of ECPRF. Anthocyanins deserve particular attention, as they have antiviral, antimicrobial, blood pressure-regulating, and anti-neurodegenerative properties [42]. These compounds also contribute to reducing the risk of cancer, cardiovascular disease, diabetes, and Alzheimer's disease [42]. It should be remembered that the amount of most of these compounds decreases during baking. Alvarez-Jubete et al. [3] clearly stated the negative influence of thermal processes on content of phenolic compounds. The drop in phenolic compounds during baking is caused by thermal degradation, enzymatic and oxidative degradation, and in the case of phenolic acids decarboxylation to 4-vinylguaiacol [18]. Therefore, the presence of such compounds in these breads indicates the importance of further research in this area. In the studies of Gumul et al. [8] regarding gluten-free bread with dried red potatoes, it has been proven that despite baking, some flavonoids and anthocyanins have been preserved. In the studies of Constantini et al. [5] concerning wheat and gluten-free breads with chia seeds, it was observed that wheat bread with chia seeds was characterized by a higher content of flavonoids than the control, while in gluten-free breads, this trend was not recorded. In the studies of Šarić et al. [43] regarding gluten-free cookies with cranberry and blueberry extracts, there was a significant increase in the content of anthocyanins after the use of strawberry pomace, compared to the control.

Starch-based breads with preparations without fruit pomace were characterized by greater antioxidant activity than control bread and the largest ones with addition of ECPRF. Antioxidant activity was higher for breads obtained with ECPRF extruded at 120°C in comparison to those containing the product processed at 80°C. It was clearly shown that the highest antioxidant activity was obtained by adding extrudates obtained at 120°C with 20% fruit pomace, which was caused by the large total polyphenol content and the high content of anthocyanins and flavonoids, because these groups of compounds generate high antioxidant activity. Also the samples, K, SBK0/80, SBK0/120 revealed antioxidant activity, because under baking conditions, the product of Maillard reaction could be formed, which further contributes to antioxidant properties of bread. This confirms earlier observations of Nicoli et al. [44] that the compounds formed at high temperatures, which exhibit antioxidant properties, may contribute to the antioxidant activity. Bioactive compounds such as carotenoids and glutathione, which have not been determined in this study, may also contribute to this activity [16]. The correlation coefficients between ABTS and TPC, flavonoids, and anthocyanins equaled 0.700, 0.700, and 0.892, respectively.

Similarly, in Gumul et al. [8] regarding gluten-free bread with dried red potatoes, the antioxidant activity of the aforementioned loaves increased rapidly in the range from five to six times in relation to the control. In the studies of Korus et al. [12] gluten-free breads with 5% and 15% share of defatted strawberry and currant seeds were characterized by greater antioxidant activity in relation to the control. However, in studies by Šarić et al. [43] concerning gluten-free cookies with cranberry and blueberry extracts, it was clearly demonstrated that the antioxidant activity of the latter is definitely higher than the first. According to the Lee and Wrolstad [45] studies on the blueberry skin, it is characterized by the highest antioxidant activity; hence, cakes with its share will have the greatest antioxidant potential.

### 3.4. Volume and Image Analysis Parameters

The volume of bread containing the applied extrudates was significantly higher compared to the control sample. Breads containing the product extruded at 120°C did not differ in volume from each other, whereas in the case of the preparation extruded at 80°C, the volume of bread was significantly different (Table 5). The increase in the volume of loaves containing extrudates could be the result of the sugars introduced with the fruit pomace as well as the depolymerization of starch during extrusion [46].

In the case of the cell/total area ratio, a small variation in the examined bread was found, although, except of the sample SBK0/80, it was higher compared to the control, which indicates their greater porosity (Table 5). This parameter showed a significant correlation with the volume (*r* = 0.82).

**Figure 1:**
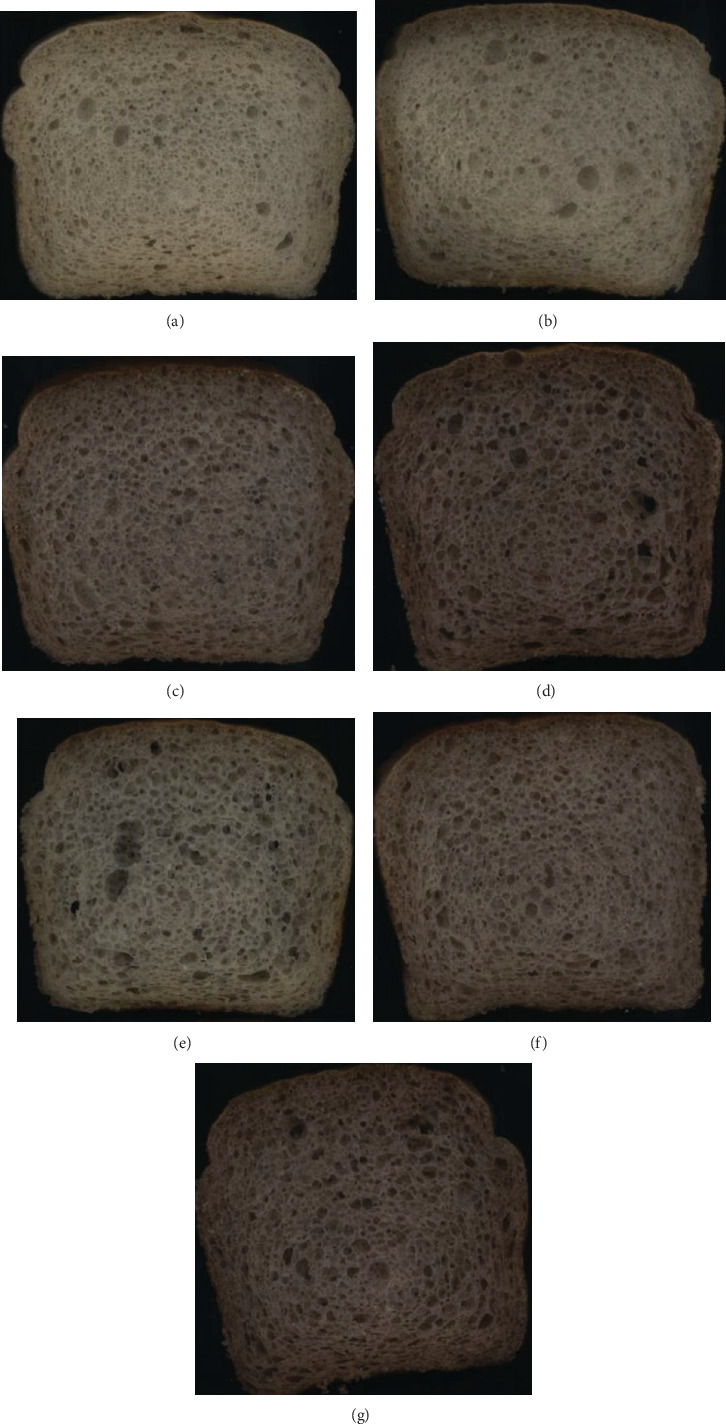
Digital images of slices of the investigated breads: (a) K control bread, (b) SBK 0/80, (c) SB10/80, (d) SB20/80, (e) SBK 0/120, (f) SB 10/120, and (g) SB 20/120. SBK 0/80, SBK 0/120, starch bread with 10% rice extrudate processed at 80 and 120°C, respectively; SB10/80, SB10/120, starch bread with 10% rice extrudate containing 10% fruit pomace, processed at 80 and 120°C, respectively; SB20/80, SB20/120, starch bread with 10% rice extrudate containing 20% fruit pomace, processed at 80 and 120°C, respectively.

The participation in the recipe of tested extrudates containing gelatinized starch caused a significant increase in both the number of large pores, over 5 mm^2^ and the average pore size, which resulted in more aerated crumb (Table 5, Figure 1). The consequence of larger pore formation was a significant decrease in cell density (the correlation coefficient *r* = −0.97 for large pores and *r* = −0.98 for average pore size). Similar observations were reported by Naito et al. [47] who added gelatinized starch to wheat bread, which resulted in an increase in pore size and reduction of their number.

### 3.5. Color

The addition of applied extrudates caused a significant darkening of the crumb of gluten-free breads compared to the control bread, which resulted from the dark color of the pomace. However, the color of bread crumb containing the same preparations but extruded at different temperatures did not differ significantly (Table 6, Figure 1).

The value of the a∗ parameter in the control bread was close to the value presented by Ziobro et al. [48]. Participation of the extrudates in the recipe increased the value of this parameter, which indicates an increasing intensity of red, which was a natural consequence of the content of red-colored pomace. In turn, the *b*∗ parameter (yellow color intensity) was significantly lower in the tested breads than in the control bread, but the variation within the samples containing the tested extrudates was smaller than in the other two parameters (Table 6). Similarly, the decrease in lightness (*L*) and yellowness (*b*∗) and the increase in redness (*a*∗) of bread crumb corresponding to increasing level of cherry powder was observed by Yoon et al. [49]. *∆E*∗ parameter indicates the differences between the color of the two samples (the larger value, the greater difference). All the bread samples differed significantly in terms of this parameter from control bread. The smallest differences were caused by the addition of the extrudates of pure rice flour, especially those processed at a lower temperature (80°C). The application of extrudates containing sour cherry pomace caused much larger differences in color in comparison to control bread, which were the larger, the greater was the share of extrudates in bread formulation.

### 3.6. Texture

**Figure 2:**
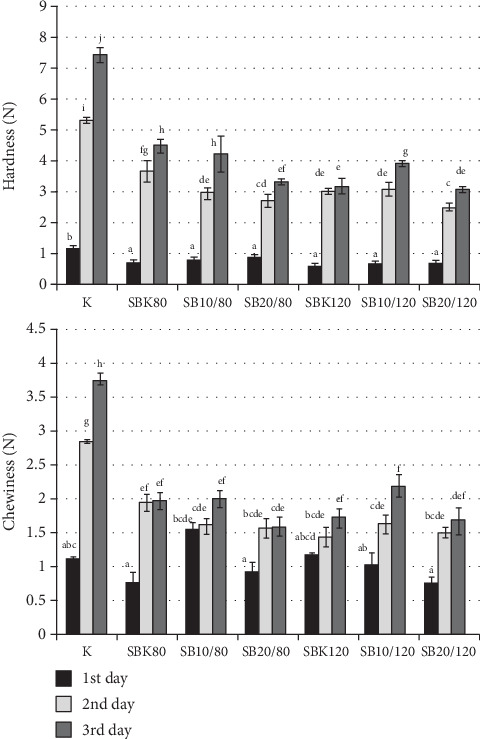
Changes in selected texture parameters of gluten-free bread within 3 days of storage: K control bread, SBK 0/80, and SBK 0/120, starch bread with 10% rice extrudate processed at 80 and 120°C, respectively; SB10/80, SB10/120, starch bread with 10% rice extrudate containing 10% fruit pomace, processed at 80 and 120°C, respectively; SB20/80, SB20/120, starch bread with 10% rice extrudate containing 20% fruit pomace, processed at 80 and 120°C, respectively (values signed with the same letters do not differ significantly at 0.05 level of confidence). Presented data are mean value of 4 replicates.

A two-factor analysis of variance showed a significant effect of the recipe, i.e., quantity of ECPRF and extrusion conditions and storage time as well as the interaction of both factors on the hardness of tested loaves. The crumb of control bread was significantly harder on each day compared to the loaves containing the extrudates (Figure 2). According to Sciarini et al. [50], the initial crumb hardness of the gluten-free bread examined by these authors was related to their volume (larger volume—lower hardness), which is confirmed in these studies. Importantly, in the majority of cases, the crumb of the loaves under study hardened more slowly than in the case of control bread. On the third day, the hardness of the bread crumb was more than 6 times higher than on the day of baking, while, *e.g.*, in the case of SB20/80 less than 4 times, SB20/120 slightly more than 4 times. This could be due to the effect of added extrudates containing fruit pulp, rich in fiber, which depending on botanical origin can bind 3-10 times more water in relation to its mass [51]. Similarly, a slight increase in the hardness of the bread crumbs containing soy flour was observed by Sciarini et al. [52], which was explained by the authors as the effect of high water absorption of soy proteins. As a result of water binding by a strongly hydrophilic component, less water is available for starch, which reduces the rate of its recrystallization during bread storage. Mechanical strength of bread crumb is affected by many factors, and the dominant effects are due to cell walls encompassing individual pores of crumb matrix [53]. Hager and Arendt [31] observed the increase of wall thickness in the crumb of analyzed gluten-free bread corresponding to the increase in water content. On the other hand, the presence of fiber could make the cells more fragile to mechanical forces.

In the case of springiness, the impact of the extrudates was negligible, while the storage time of the bread and the interaction of both factors were important. In general, the springiness of bread crumb decreased during storage, but the differences were not large, although as indicated, statistically significant (data not shown). The cohesiveness of bread crumb was affected only by the time of storage (data not shown).

The chewiness of gluten-free breads with the addition of extrudates did not differ significantly on the day of baking from the control bread, as well as in comparison to each other (the exception was the higher value of this parameter in SB10/80). However, on the second and third day of storage, the chewiness of the control bread was significantly higher than of the breads containing extrudates (Figure 1).

## 4. Conclusions

Summing up, the extrudates with sour cherry pomace enrich gluten-free bread in bioactive compounds. The best results were obtained when using the extrudates with 20% sour cherry pomace obtained at 120°C. Bread with the addition of the preparation 20/120 was the most favorable in terms of bioactive compounds (TPC, flavonoids, anthocyanins, and phenolic acids) and antioxidative activity. It also contained the highest amount of total, soluble, and insoluble fiber, and a significant content of ash and sugars.

The 20/120 preparation used for baking gluten-free breads contributed to a delay in crumb hardening during bread storage in comparison with the control and other samples. At the same time, it did not deteriorate other texture parameters. The preparation could be recommended as a potential component for industrial production of gluten-free bread.

## Figures and Tables

**Table 1:** Chemical composition of analyzed bread samples.

Sample	Protein (g/kg d.m.)	Fat (g/kg d.m.)	Ash (g/kg d.m.)	Sugars (g/kg d.m.)	Starch (g/kg d.m.)	Moisture (g/kg)
K	23.3 ± 1.1^a^	44.9 ± 0.2^cd^	18.5 ± 0.4^a^	0.9 ± 0.1^a^	736.2 ± 3.5^e^	489.5 ± 1.8^a^
SBK 0/80	24.2 ± 1.0^a^	42.6 ± 1.7^c^	18.4 ± 0.1^a^	6.9 ± 0.2^c^	733.8 ± 1.7^e^	512.5 ± 2.4^b^
SB 10/80	24.3 ± 0.4^a^	41.4 ± 1.1^c^	18.5 ± 0.2^a^	6.1 ± 0.2^b^	718.3 ± 1.2^c^	514.1 ± 2.1^b^
SB 20/80	25.5 ± 0.2^a^	36.9 ± 0.6^b^	20.6 ± 0.1^b^	6.3 ± 0.1^b^	718.3 ± 2.5^c^	515.8 ± 3.1^b^
SBK 0/120	24.0 ± 0.4^a^	31.0 ± 2.0^a^	18.7 ± 0.2^a^	6.0 ± 0.1^b^	724.8 ± 1.2^d^	520.7 ± 2.9^c^
SB 10/120	24.0 ± 0.1^a^	38.7 ± 1.1^b^	20.2 ± 0.4^b^	7.1 ± 0.1^c^	712.6 ± 3.0^b^	524.3 ± 3.2^c^
SB 20/120	25.5 ± 0.4^a^	41.0 ± 0.6^c^	20.2 ± 0.2^b^	6.8 ± 0.2^c^	695.2 ± 1.8^a^	525.0 ± 3.5^c^

Presented data are mean values (*n* = 2) ± standard deviation (values signed with the same letters in particular columns do not differ significantly at 0.05 level of confidence). K control bread, SBK 0/80, and SBK 0/120, starch bread with 10% rice extrudate processed at 80 and 120°C, respectively; SB10/80, SB10/120, starch bread with 10% rice extrudate containing 10% fruit pomace, processed at 80 and 120°C, respectively; SB20/80, SB20/120, starch bread with 10% rice extrudate containing 20% fruit pomace, processed at 80 and 120°C, respectively. d.m.: dry matter.

**Table 2:** Content of dietary fiber in analyzed bread sample (in dry matter).

Sample	Insoluble (g/kg)	Soluble (g/kg)	Total (g/kg)
K	34.0 ± 1.0^d^	17.6 ± 0.7^a^	51.6 ± 0.3^b^
SBK 0/80	25.9 ± 0.2^a^	21.8 ± 0.1^b^	47.7 ± 0.2^a^
SB 10/80	31.6 ± 0.6^b^	21.8 ± 0.3^b^	53.4 ± 0.4^c^
SB 20/80	33.7 ± 0.8^c^	21.8 ± 0.4^b^	55.5 ± 0.4^d^
SBK 0/120	25.1 ± 0.9^a^	23.4 ± 0.6^d^	48.5 ± 0.3^a^
SB 10/120	34.5 ± 0.7^d^	22.9 ± 0.1^c^	57.4 ± 0.5^e^
SB 20/120	35.6 ± 0.6^d^	23.9 ± 0.7^d^	59.5 ± 1.3^f^

Presented data are mean values (*n* = 2) ± standard deviation (values signed with the same letters in particular columns do not differ significantly at 0.05 level of confidence). Abbreviations of samples are the same as explained in footnote of Table 1.

**Table 3:** Antioxidant activity and total polyphenol content in the examined bread samples (in dry matter).

Sample	Antioxidant activity (*μ*M Tx/kg)	Total polyphenol content (TPC) (mg catechin/kg)
K	0.379 ± 0.021^b^	Not detected
SBK 0/80	1.408 ± 0.050^c^	3.1 ± 0.5^a^
SB 10/80	1.817 ± 0.017^d^	59.4 ± 1.1^b^
SB 20/80	2.137 ± 0.000^e^	65.8 ± 0.8^c^
SBK 0/120	0.221 ± 0.000^s^	Not detected
SB 10/120	2.377 ± 0.079^f^	136.1 ± 1.3^d^
SB 20/120	2.297 ± 0.052^f^	308.7 ± 0.7^e^

Presented data are mean values (*n* = 4) ± standard deviation (values signed with the same letters in particular columns do not differ significantly at 0.05 level of confidence). Abbreviations of samples are the same as explained in footnote of Table 1.

**Table 4:** Contents of flavonoids, anthocyanins, and phenolic acids in the examined bread samples (in dry matter).

Sample	Flavonoids (mg rutin/kg)	Anthocyanins (mg cyanidin-3-glucoside/kg)	Phenolic acids (mg ferulic acid/kg)
K	Not detected	Not detected	Not detected
SBK 0/80	6.4 ± 2.1^a^	Not detected	Not detected
SB 10/80	11.7 ± 2.4^b^	26.1 ± 2.0^a^	Not detected
SB 20/80	17.8 ± 1.3^c^	28.3 ± 1.0^a^	Not detected
SBK 0/120	Not detected	Not detected	Not detected
SB 10/120	57.4 ± 1.1^d^	27.4 ± 1.3^a^	2.14 ± 0.20^a^
SB 20/120	97.3 ± 2.8^e^	34.2 ± 1.2^b^	2.37 ± 0.00^b^

Presented data are mean values (*n* = 4) ± standard deviation (values signed with the same letters in particular columns do not differ significantly at 0.05 level of confidence). Abbreviations of samples are the same as explained in footnote of Table 1.

**Table 5:** Volume and image analysis parameters of analyzed bread samples.

Sample	Volume (mL)	Mean cell area (mm^2^)	Cell density (cells/cm^2^)	Pores > 5mm^2^ (%)	Cell/total area
K	495 ± 4^a^	1.09 ± 0.03^a^	41 ± 1^d^	4.4 ± 0.6^a^	0.45 ± 0.00^a^
SBK 0/80	520 ± 8^b^	1.55 ± 0.02^b^	30 ± 1^c^	8.4 ± 0.3^b^	0.46 ± 0.01^a^
SB 10/80	531 ± 8^c^	1.84 ± 0.12^c^	27 ± 2^b^	11.1 ± 0.9^c^	0.49 ± 0.01^c^
SB 20/80	562 ± 9^d^	1.90 ± 0.20^c^	26 ± 2^b^	11.3 ± 1.6^c^	0.50 ± 0.01^c^
SBK 0/120	523 ± 9^bc^	2.23 ± 0.15^d^	22 ± 1^a^	14.7 ± 1.3^d^	0.48 ± 0.01^b^
SB 10/120	521 ± 7^bc^	1.77 ± 0.05^c^	27 ± 1^b^	9.8 ± 0.6^bc^	0.48 ± 0.01^b^
SB 20/120	527 ± 3^bc^	1.79 ± 0.14^c^	27 ± 2^b^	10.9 ± 1.8^c^	0.48 ± 0.01^b^

Presented data are mean values (*n* = 6) ± standard deviation (values signed with the same letters in particular columns do not differ significantly at 0.05 level of confidence). Abbreviations of samples are the same as explained in footnote of Table 1.

**Table 6:** Color parameters of analyzed bread samples.

Sample	*L*∗	*a*∗	*b*∗	*∆E*∗
K	72.69 ± 0.97^e^	−0.98 ± 0.16^a^	15.11 ± 0.57^d^	—
SBK 0/80	70.15 ± 1.00^d^	−1.07 ± 0.08^a^	13.96 ± 0.00^c^	2.81 ± 0.21^a^
SB 10/80	63.44 ± 0.70^bc^	2.17 ± 0.06^b^	12.42 ± 0.37^b^	10.16 ± 0.06^c^
SB 20/80	57.12 ± 1.17^a^	4.35 ± 0.25^d^	12.10 ± 0.04^ab^	16.73 ± 0.31^e^
SBK 0/120	64.87 ± 0.40^c^	−1.25 ± 0.10^a^	12.75 ± 0.33^b^	8.17 ± 0.47^b^
SB 10/120	62.67 ± 0.25^b^	2.51 ± 0.24^b^	12.52 ± 0.67^b^	10.92 ± 0.49^d^
SB 20/120	57.57 ± 0.88^a^	3.93 ± 0.03^c^	11.42 ± 0.05^a^	16.32 ± 0.00^e^

Presented data are mean values (*n* = 6) ± standard deviation (values signed with the same letters in particular columns do not differ significantly at 0.05 level of confidence). Abbreviations of samples are the same as explained in footnote of Table 1.

## Data Availability

The data used to support the findings of this study are included within the article.
